# Targeting tumor-infiltrating regulatory T cells: combining CD47 and PD-L1 inhibition via a novel aptamer-siRNA chimera

**DOI:** 10.1186/s43556-025-00392-2

**Published:** 2025-12-17

**Authors:** Yu Zeng, Xiaoli Chen, Wenqiong Huang, Chi Ho Chan, Ziqi Chen, Minchuan Lyu, Yumeng Liu, Meijun Liu, Aiping Lyu, Claudio Mauro, Yuanyuan Yu, Kenneth CP Cheung

**Affiliations:** 1 Phenome Research Center, School of Chinese Medicine, Hong Kong Baptist University, Hong Kong, China; 2 Institute of Integrated Bioinformedicine and Translational Science (IBTS), School of Chinese Medicine, Hong Kong Baptist University, Hong Kong, China; 3 Guangdong-Hong Kong-Macao Greater Bay Area International Research Platform for Aptamer-Based Translational Medicine and Drug Discovery, Hong Kong, China; 4 College of Medicine and Dental Sciences, University of Birmingham, Birmingham, UK

**Keywords:** Regulatory T cells, CD47, PD-L1, Aptamer-siRNA chimera, Tumor microenvironment, Immunotherapy

## Abstract

Tumor-infiltrating regulatory T (Treg) cells contribute to immune evasion and are associated with poor prognosis in solid tumors. While CD47 blockade has demonstrated efficacy in hematologic malignancies, its application in solid tumors is hindered by the antigen sink effect and lack of tumor selectivity. Here, we report a rationally designed aptamer-siRNA chimera that selectively targets intratumoral Treg cells by exploiting their co-expression of PD-L1 and CD47 within the tumor microenvironment. The PD-L1 aptamer enables selective binding to PD-L1⁺ Treg cells and simultaneously inhibits PD-L1-mediated immune suppression. Conjugated CD47 siRNA silences CD47 expression, abrogating the “don’t eat me” signal and facilitating phagocytic clearance. Mechanistically, this chimera efficiently depletes tumor-infiltrating Treg cells with negligible impact on peripheral cells, and leads to a pronounced increase in intratumoral CD8⁺ T cell infiltration. Further investigation revealed that the chimera impairs Treg migration by disrupting glycolysis-related signaling pathways, including pERK1/2 and pRac1, and induces metabolic reprogramming characterized by reduced glycolysis, increased oxidative metabolism, and elevated fatty acid oxidation (FAO). In murine hepatocellular carcinoma models, treatment with the chimera significantly inhibited tumor growth, reduced angiogenesis, and prolonged survival. Our findings highlight a dual immune checkpoint-targeting strategy that integrates selective delivery with gene silencing, offering a tumor-specific, non-antibody approach for Treg depletion and a promising avenue for solid tumor immunotherapy.

**Supplementary Information:**

The online version contains supplementary material available at 10.1186/s43556-025-00392-2.

## Introduction

Emerging research underscores the therapeutic potential of targeting the signal-regulatory protein alpha (SIRPα)-CD47 immune checkpoint in cancer treatment [1, 2]. CD47 is often overexpressed on tumor cells, where it binds to SIRPα on macrophages to transmit an inhibitory “do not eat me” signal that suppresses phagocytosis and promotes immune evasion [3–5]. Elevated CD47 expression has been observed across a range of human and murine tumors. Preclinical studies have demonstrated that CD47 blockade induces robust antitumor responses particularly in hematologic malignancies [6, 7], and its therapeutic efficacy is enhanced when combined with tumor-targeting antibodies such as rituximab, which engage activating Fc receptors to deliver pro-phagocytic “eat me” signals [8, 9]. However, major obstacles, such as the CD47 antigen sink and limited efficacy in solid tumors, remain to be overcome [10, 11]. To address these limitations, recent strategies have shifted toward modifying the SIRPα/CD47 axis to enhance macrophage-mediated clearance, often in combination with other immunomodulatory approaches [12].

In parallel, accumulating evidence has indicated that Treg cells drive tumor immune evasion in solid tumors, particularly in hepatocellular carcinoma (HCC) [13]. Notably, tumor-infiltrating Treg cells are characterized by high expression of immune checkpoint molecules, including PD-L1 and CD47, which contribute to their immunosuppressive function in the tumor microenvironment (TME) [14–16]. However, while systemic inhibition of these checkpoints has shown therapeutic promise, such approaches are frequently hampered by dose-limiting toxicities arising from off-tumor effects [17]. Thus, there is a critical need for strategies that enable selective targeting of intratumoral Tregs while sparing peripheral cells.

To overcome these challenges, we introduce a novel dual-targeting RNA therapeutic: a PD-L1-binding aptamer conjugated with a CD47-specific siRNA. Compared with conventional antibodies, aptamers possess high selectivity, low immunogenicity, and facile synthesis, enabling precise delivery of therapeutic payloads to specific cell populations [18, 19]. Leveraging the unique co-expression of PD-L1 and CD47 on tumor-infiltrating Tregs, our aptamer-siRNA chimera achieves cell-type-selective delivery of RNA interference, combining immune checkpoint blockade with gene silencing in a single molecule. The design offers multiple advantages: 1) preferential accumulation in PD-L1⁺ Treg cells within the TME; 2) avoidance of nonspecific uptake by peripheral blood cells such as red blood cells (RBCs) or splenic Tregs; and 3) potential synergy from simultaneous inhibition of PD-L1 and CD47 signaling pathways.

Here, we hypothesized that simultaneously targeting PD-L1 and CD47 on tumor-infiltrating Treg cells could result in their depletion, metabolic reprogramming, and enhanced antitumor immunity—while reducing off-tumor toxicity. To test this hypothesis, we developed the PD-L1-targeting aptamer-CD47 siRNA chimera, and evaluated its efficacy in a murine HCC model. Our investigations demonstrate that this approach simultaneously inhibited PD-L1-mediated immunosuppressive signaling and silenced CD47 expression, thereby suppressing tumor growth, induces selective depletion and metabolic alteration of Tregs, and boosts CD8⁺ T cell infiltration, establishing aptamer-siRNA conjugates as a platform for effective RNAi-based immunomodulation within the TME.

## Results

### PD-L1-targeting aptamer-siRNA chimera selectively targets intratumoral Treg cells and induces effective CD47 silencing

To elucidate the mechanisms underlying tumour immune evasion and guide the design of targeted interventions, we first performed comprehensive pathway enrichment and network analyses focused on genes functionally associated with PD-L1 and SIRPα/CD47 signalling. Candidate genes including IL2, IFNG, TNF, TBX21, EOMES, CTLA4, LAG3, BCL2, BAX, GLUT1, HK2, IL6, IL12, IL10, TGF-β, RAC1, and mTOR—were selected using KEGG and Curated Elsevier Pathway Collection databases with an FDR cutoff of 0.05.

STRING-based protein–protein interaction (PPI) network analysis was conducted using ShinyGO 0.81, which revealed strong associations with key immune-related pathways, including T cell receptor signaling, Th1/Th2/Th17 differentiation, and NK cell-mediated cytotoxicity. In addition, enrichment analysis identified links to metabolic pathways such as glycolysis and fatty acid metabolism, as well as pathways involved in cancer immune evasion (e.g., IDO1 activity and effector T cell suppression) (Fig. S1a-d).

**Fig. 1:**
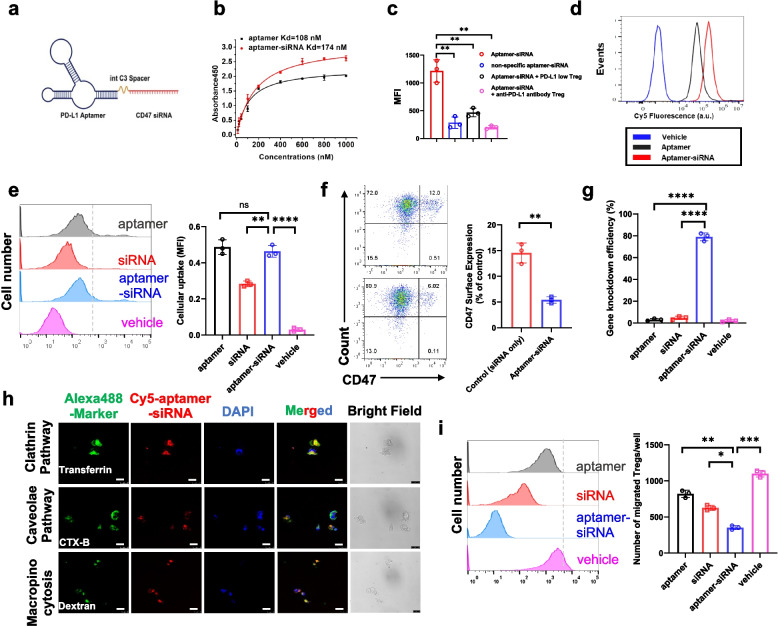
Synthesis and characterization of aptamer-siRNA targets intratumoral Treg cells. **a** Schematic illustration of the PD-L1 aptamer conjugated to CD47 siRNA via an internal C3 spacer linker. **b** Binding affinities of the aptamer and aptamer-siRNA conjugate to recombinant human PD-L1 protein were measured by enzyme-linked oligonucleotide assay (ELONA). **c** Cy5-labelled aptamer-siRNA binding to tumour-infiltrating Treg cells isolated from Hepa1 HCC mouse tumours under the following conditions: incubation with aptamer-siRNA, non-specific aptamer-siRNA, aptamer-siRNA in PD-L1 low Treg cells, or aptamer-siRNA following anti-PD-L1 antibody pre-treatment. **d** Binding of Cy5-labelled aptamer and aptamer-siRNA to tumor-infiltrating Treg cells was evaluated by flow cytometry. **e** Cellular uptake of aptamer-siRNA conjugate by tumor-associated Treg cells was quantified by fluorescence intensity. **f** Flow cytometry analysis of CD47 surface protein expression on tumor-infiltrating Treg cells following treatment with siRNA or aptamer-siRNA. **g** CD47 knockdown efficiency in Treg cells was quantified by qPCR following the under indicated treatments. **h** Representative confocal images illustrating intracellular uptake pathway by co-localization of Cy5-labelled aptamer-siRNA (red) with Alexa Fluor 488-labelled endocytic pathway markers (transferrin, cholera toxin and dextran; green) in tumor-infiltrating Treg cells; nuclei are counterstained with DAPI (blue). Scale bars, 25 μm. **i** In vitro transmigration of tumor-associated Treg cells following treatment with the aptamer-siRNA conjugate. Results are expressed as mean ± s.d., with three independent samples measured in triplicate for each experiment. *P < 0.05, **P < 0.01, ***P < 0.001, ****P < 0.0001

We next sought to experimentally test this hypothesis by rationally designing a bifunctional aptamer-siRNA chimera capable of simultaneously engaging PD-L1 and silencing CD47. Specifically, a PD-L1-targeting aptamer previously developed by our group [20], was conjugated to a CD47-specific siRNA via an internal C3 spacer, resulting in the formation of the aptamer-siRNA chimera (Fig. 1a). We then evaluated the binding affinity of the aptamer-siRNA conjugate to recombinant human PD-L1 protein using enzyme-linked oligonucleotide assay (ELONA). The conjugation of CD47 siRNA resulted in a moderate reduction in binding affinity, with the Kd increasing from 108nM (aptamer alone) to 174nM (aptamer-siRNA conjugate) (Fig. 1b). Despite this reduction, the conjugate maintained nanomolar-range affinity, which is much lower than the sub-nanomolar affinities typically exhibited by high-affinity antibodies. This decreased affinity was design to mitigate off-target interactions with PD-L1-expressing peripheral cells such as RBCs and systemic Treg cells, while enabling selective recognition of PD-L1⁺ Treg cells within the TME [21]. To validate he PD-L1 binding specificity, we assessed its binding to tumor-infiltrating Treg cells isolated from Hepa1-6-induced HCC tumor bearing mice. The chimera exhibited robust binding to Treg cells expressing high levels of PD-L1, in contrast, minimal binding was observed when cells were pre-blocked with anti-PD-L1 antibody or in PD-L1low cells, confirming selectivity for high PD-L1 expression (Fig. 1c). Furthermore, Flow cytometry confirmed comparable binding of the aptamer-siRNA conjugate and free aptamer to tumor-associated Treg cells, demonstrating that the conjugate preserves cell-binding activity (Fig. 1d).

To investigate the internalization of aptamer-siRNA by tumor-associated Treg cells, cells were incubated with Cy5-labeled aptamer-siRNA, with free aptamer and siRNA as controls. Quantification of intracellular fluorescence showed that uptake of the aptamer-siRNA conjugate was significantly higher than that of free siRNA and comparable to that of free aptamer (Fig. 1e). Meanwhile, aptamer-siRNA enabled efficient targeted delivery and gene silencing, resulting in robust CD47 knockdown and decreased CD47 surface expression in tumor-infiltrating Treg cells, as confirmed by qPCR and flow cytometry (Fig. 1f-g). Notably, free siRNA showed a moderate fluorescence signal, likely due to non-specific adsorption or limited endocytic uptake [22]. However, inefficient cytoplasmic delivery prevented effective CD47 knockdown.

To elucidate the pathway underlying cellular uptake of the aptamer-siRNA, we subsequently labeled the conjugate with Cy5 and performed confocal microscopy in the presence of Alexa Fluor 488-labeled endocytic markers. Co-localization with transferrin (clathrin-mediated endocytosis marker), choleratoxin (caveolae-mediated endocytosis marker), and dextran (micropinocytosis marker) suggested that the aptamer-siRNA conjugate is predominantly internalized via the clathrin-mediated pathway (Fig. 1h). In vitro, aptamer-siRNA-treated Treg cells exhibited impaired chemotaxis toward IP10 (CXCL10), a chemokine relevant to liver TME, suggesting that the aptamer-siRNA was effectively internalized and functionally active in disrupting Treg trafficking (Fig. 1i). Altogether, these results characterize the aptamer-siRNA chimera as capable of selectively binding to tumor-infiltrating Treg cells, being efficiently internalized, and inducing effective CD47 knockdown.

### In vivo biodistribution and safety evaluation of dual-targeting PD-L1 × CD47 siRNA.

**Fig. 2:**
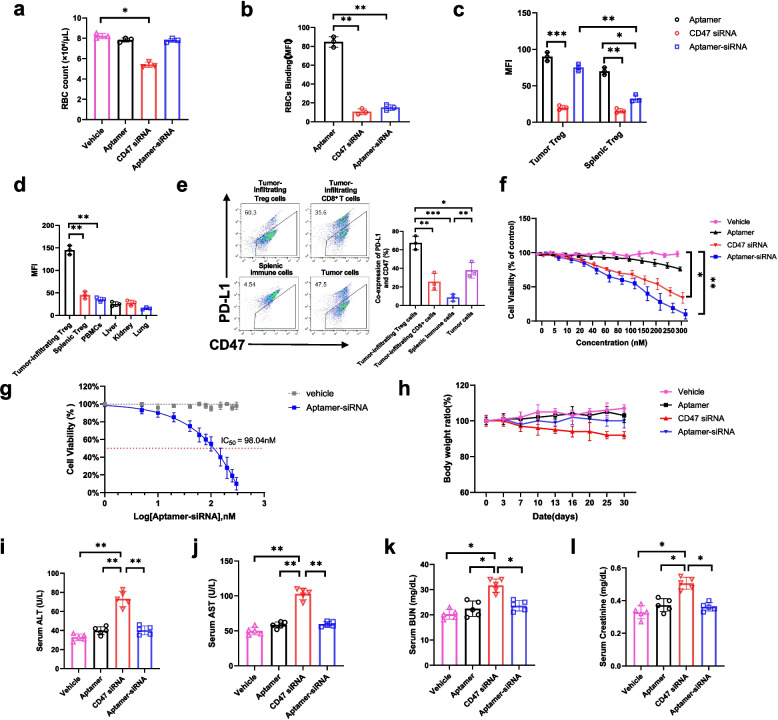
Tumor targeting specificity and in vivo safety profile of the aptamer siRNA chimera. **a-b** RBC count and RBC binding of Cy5-labeled PD-L1 aptamer, CD47 siRNA, and PD-L1 × CD47 siRNA. **c** Comparison of in vitro uptake by tumor-infiltrating Treg cells and splenic Treg cells across all groups. **d** In vivo biodistribution analysis after intravenous injection of Cy5-labeled aptamer-siRNA in tumor-bearing mice. Fluorescence intensity in tumor-infiltrating Treg cells, splenic Treg cells, PBMCs, liver, kidney, and lung were measured. **e** Flow cytometry analysis of PD-L1 + CD47 + co-expression in various cell populations within the TME. **f-g** The viability and IC50 of tumor-infiltrating Treg cells following treatment with the aptamer-siRNA conjugate were measured by MTT assay. **h** Body weight of healthy mice monitored for 30 days after administration of different treatments. **i-l** Serum levels of liver function markers (AST, ALT) and kidney function markers (BUN, creatinine) were measured by ELISA in healthy mice following different treatments. Data are shown as mean ± s.d. (n = 5 per group). Statistical analysis was determined by one-way ANOVA with Tukey’s post hoc test. *P < 0.05, **P < 0.01, ***P < 0.001, ****P < 0.0001

To evaluate the in vivo safety profile of the aptamer-siRNA chimera, C57BL/6J mice were administered vehicle, aptamer, siRNA, or the aptamer-siRNA chimera. Subsequent analysis of RBC counts revealed a slight decrease in the siRNA-treated group, while counts in the aptamer-siRNA chimera group were comparable to those in controls (Fig. 2a). Further assays using isolated RBCs and splenic Treg cells demonstrated that the conjugate exhibited very low binding to RBCs and splenic Treg cells while showed significantly increased uptake by tumor-infiltrating Treg cells (Fig. 2b-c). Consistently, biodistribution analysis showed that the aptamer-siRNA exhibited significantly higher accumulation in tumor-infiltrating Treg cells compared to splenic Treg cells and peripheral blood mononuclear cells (PBMCs), with minimal signals in liver, kidney, and lung tissues (Fig. 2d). Additionally, flow cytometry analysis of PD-L1 and CD47 co-expression in various cell populations within the TME, along with splenic immune cells as controls, revealed that tumor-infiltrating Treg cells exhibited the highest levels of PD-L1 + CD47 + co-expression (Fig. 2e).

To further characterize the biological activity of the aptamer-siRNA chimera at the cellular level, we performed MTT assays, which revealed an IC50 value of 98.04 nM, demonstrating potent inhibitory effects on target cells (Fig. 2f-g). Subsequently, in vivo organ toxicity was evaluated by longitudinal body weight monitoring and comprehensive liver and kidney function assessments. Serum AST and ALT levels served as indicators of hepatocellular injury, while blood urea nitrogen (BUN) and creatinine levels were measured to assess renal function. Our results showed that no significant differences in body weight were observed among treatment groups, indicating an absence of systemic toxicity (Fig. 2h). Furthermore, enzyme levels and renal function markers in chimera-treated mice were comparable to vehicle controls, suggesting minimal hepatotoxicity and nephrotoxicity (Fig. 2i-l). Collectively, these results demonstrate that the PD-L1 × CD47 siRNA conjugate exhibits strong tumor-targeting specificity and peripheral selectivity, preferentially accumulating in tumor-infiltrating Treg cells while sparing systemic immune cells and major organs. This selective biodistribution supports the favorable safety profile of the aptamer-siRNA chimera and highlights its therapeutic potential without inducing detectable liver or kidney damage.

### Combined inhibition of PD-L1 and CD47 signals significantly inhibits liver cancer growth

**Fig. 3:**
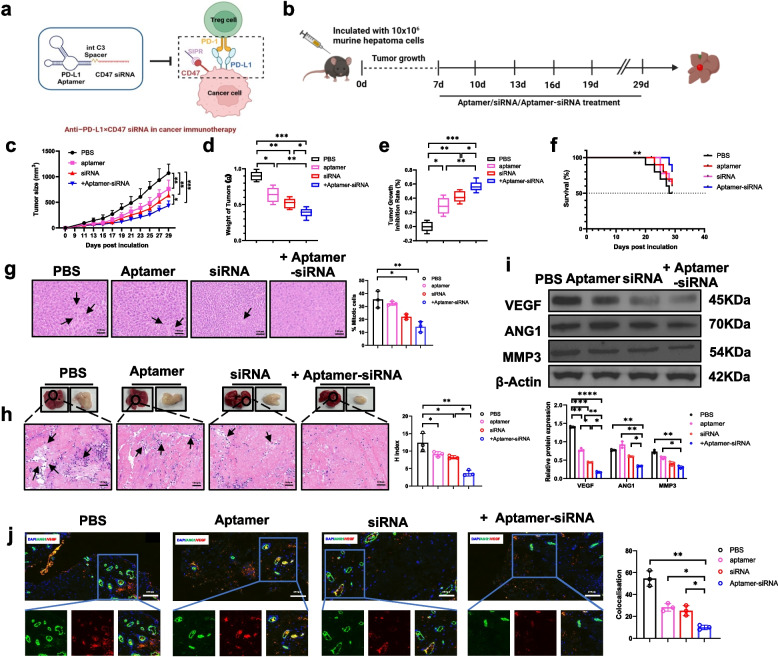
Anti-PD-L1 × CD47 siRNA reduces tumor growth and angiogenesis in HCC. **a** Schematic diagram of PD-L1 and CD47 signaling pathways in cancer immunotherapy. **b** Schematic illustrating the experimental design: C57BL/6J female mice (n = 10 per group) were subcutaneously inoculated with 10 × 10.^6^ murine hepatoma cells on day 0 to induce tumor formation. Starting from day 7, mice were received intravenous injections of anti-PD-L1 aptamer, CD47 siRNA, PD-L1 × CD47 siRNA chimera (2 mg/kg) or vehicle every 3 days until day 29. **c** Tumor growth was monitored over time in four groups: PBS, aptamer, siRNA, and aptamer-siRNA conjugate. **d** Weight of tumor tissues in each group at the end of treatment. **e** Tumor inhibition rate of mice after different treatments. **f** Kaplan–Meier survival analysis shows significantly prolonged survival following aptamer-siRNA treatment. **g** Representative H&E-stained sections of HCC tissues from each treatment group (PBS, aptamer, siRNA, aptamer-siRNA) collected 8 weeks post-treatment (scale bar, 100 μm; inset, 200 × magnification). Mitotic figures were quantified by counting 500 cells per sample. **h** Tumour growth and histopathological changes following aptamer-siRNA treatment in liver cancer models, the bar graph on the right shows the H-score quantification. Scale bar, 100µm. **i** Western blot analysis of VEGF, MMP-3, and ANG-1 in subcutaneous tumour tissues. **j** Confocal microscopy reveals a low proportion of cells expressing ANG1 and VEGF following aptamer-siRNA treatment. Quantification was performed on 500 cells per sample. Scale bar, 200 μm. Data are presented as mean ± s.d. from two or three independent experiments. Statistical significance was determined by two-way ANOVA with Tukey’s multiple comparisons test or one-way ANOVA with Tukey’s post hoc test. *P < 0.05, **P < 0.01, ***P < 0.001, ****P < 0.0001

To assess the therapeutic potential of dual PD-L1 and CD47 inhibition in HCC, we established subcutaneous Hepa 1–6 tumors in mice and treated them with anti-PD-L1 aptamer, CD47 siRNA and PD-L1 × CD47 siRNA chimera (2 mg/kg, every 3 days from day 7 to 29) (Fig. 3a-b). Treatment with the aptamer-siRNA chimera led to pronounced tumor regression, as reflected by substantial reductions in tumor size and sustained inhibition of tumor growth throughout the study period (Fig. 3c-e). Moreover, Kaplan–Meier survival analysis revealed a marked improvement in overall survival among chimera-treated mice, underscoring its therapeutic promise (Fig. 3f).

Further analyses revealed that chimera treatment resulted in decreased proliferation and mitotic activity, as indicated by reduced Ki67 staining and fewer mitotic cells in H&E-stained sections, together with increased apoptosis, demonstrated by enhanced TUNEL positivity under fluorescence microscopy (Fig. 3g and Fig. S2b-c). Quantitative histopathological evaluation confirmed a decrease histological scoring (including assessments of necrosis, mitotic index, and tumor area), indicating effective attenuation of tumor burden (Fig. 3h). Treatment with the chimera also suppressed tumor angiogenesis, evidenced by reduced expression of key angiogenic mediators, including VEGF, MMP-3, and ANG-1 (Fig. 3i and Fig. S2a). Furthermore, VEGF and ANG-1 colocalization was markedly reduced, suggesting disrupted coordinated activity within the TME (Fig. 3j). Collectively, these data demonstrate that simultaneous targeting of PD-L1 and CD47 elicits robust antitumor responses, characterized by inhibition of tumor growth, enhanced apoptosis, and impaired angiogenesis, while monotherapies achieved only limited efficacy.

### PD-L1 × CD47 siRNA chimera depletes intratumoral Treg cells and promotes CD8 + T cell infiltration

**Fig. 4:**
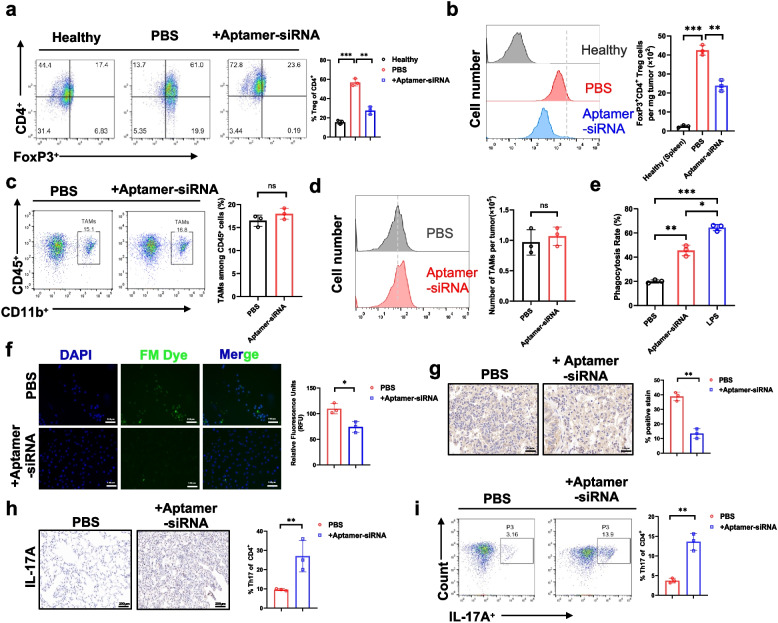
The anti-tumor activity of the PD-L1 × CD47 siRNA chimera is associated with selective depletion of intratumoral Treg cells and enhancement of Th17 responses**. a** Flow cytometric analysis of tumour-infiltrating lymphocytes showing a significant reduction in the proportion of Treg cells in the aptamer-siRNA treatment group compared to the PBS group. **b** Flow cytometry-based quantification of FoxP3⁺CD4⁺ Treg cells per milligram of tumor tissue. Tumor-infiltrating lymphocytes were isolated from subcutaneous Hepa1-6 tumors of mice treated with PBS or aptamer-siRNA, and the absolute number of FoxP3⁺CD4⁺ Treg cells was normalized to the corresponding tumor weight. **c-d** Flow cytometry analysis of tumor-associated macrophages (TAMs). Single-cell suspensions from tumors were stained with anti-CD45, anti-CD11b, and anti-F4/80 antibodies. (**c**) Absolute number of TAMs per tumor. (d) Percentage of TAMs among CD45⁺ cells. **e** Quantification of macrophage phagocytosis rates following treatment with PBS and aptamer-siRNA conjugate. LPS was used as a positive control. **f** Representative fluorescence microscopy images demonstrating decreased Treg cell infiltration in tumours after aptamer-siRNA treatment. Scale bar, 100 μm. **g** Immunohistochemistry for Foxp3 + cells showing reduced numbers of Foxp3 + nuclei in the aptamer-siRNA group versus PBS controls. Quantification was performed on 30,000 cells per section. **h** Immunohistochemical analysis confirming increased Th17 cell infiltration in tumour tissues after treatment with aptamer-siRNA. Scale bar, 200 μm. **i** Quantification of Th17 cells by flow cytometry showing a significant increase in the aptamer-siRNA treatment group. Data represent the mean ± s.d. from three independent experiments. Statistical significance was determined by one-way ANOVA with Tukey’s post hoc test or two-tailed Student’s t-test, as appropriate; ns, not significant, *P < 0.05, **P < 0.01, ***P < 0.001

To further elucidate the mechanism underlying the antitumor effect of the PD-L1 × CD47 aptamer-siRNA chimera, we analyzed immune cells composition within the TME. Flow cytometry analysis showed that chimera treatment exhibited a significant reduction in both the frequency and absolute number of FoxP3⁺CD4⁺ Treg cells per milligram of tumor tissue compared to the PBS control group (Fig. 4a-b). Subsequent analysis revealed that the total number of intratumoral CD4⁺ T cells remained unchanged; however, a marked increase was observed in the absolute number of Th17 (IL-17A⁺CD4⁺) cells, accompanied by a significant decrease in Treg cells (Fig. S3a). This change reflects a selective alteration within CD4⁺ T cell subsets, specifically characterized by Treg depletion and a compensatory increase in Th17 cells, rather than a broad shift in overall CD4⁺ T cell populations.

Quantitative flow cytometric analysis of tumor-associated macrophages (TAMs) revealed no significant differences in either the absolute number of TAMs per tumor or their proportion among CD45⁺ cells between chimera-treated and control groups (Fig. 4c-d). Similarly, expression levels of macrophage polarization markers CD80 (M1) and CD206 (M2) remained unchanged following treatment (Fig. S3b). However, assessment of phagocytic activity demonstrated that the PD-L1 × CD47 aptamer-siRNA chimera significantly enhanced macrophage phagocytosis compared to controls, suggesting that the immunomodulatory effects of the chimera are predominantly mediated through increased phagocytic function rather than alterations in TAM abundance or polarization (Fig. 4e). Given the pivotal role of CD8⁺ T cells in mediating antitumor immune responses and their regulation by Tregs, we further assessed the abundance and activation status of tumor-infiltrating CD8⁺ T cells by flow cytometry. Chimera-treated mice demonstrated a marked increase in both the frequency and absolute number of CD8⁺ T cells. Furthermore, these CD8⁺ T cells exhibited enhanced effector function, as indicated by elevated expression levels of IFN-γ, Granzyme B, and the activation marker CD69 (Fig. S3c-g). In contrast, the frequencies of monocytes and dendritic cells within the tumor tissue remained unchanged following treatment (Fig. S3h-k), demonstrating that the PD-L1 × CD47 aptamer-siRNA chimera primarily exerts its immunological effects through selective depletion of Treg cells, enhanced effector function of CD8⁺ T cells, and increased phagocytic capacity of macrophages.

To visualize the overall distribution of infiltrating lymphocytes within tumor sections, we used FM 1-43FX dye, a fixable lipophilic styryl dye that selectively stains cellular membranes and is commonly used to track immune cell populations in tissue. Analysis revealed that aptamer-siRNA-treated tumors exhibited significantly reduced FM dye fluorescence compared to PBS controls, indicative of diminished Treg cell infiltration (Fig. 4f), a finding further corroborated by immunohistochemical staining (Fig. 4g). Of note, consistent with the preceding findings, both flow cytometry and immunohistochemistry demonstrated a pronounced increase in the proportion of tumor-infiltrating Th17 cells within the CD4⁺ T cell population in chimera-treated tumors relative to PBS controls (Fig. 4h-i). This shift in the Treg/Th17 balance suggests that depletion of immunosuppressive Treg cells may relieve suppression of Th17 differentiation or expansion, thereby promoting a more pro-inflammatory and potentially anti-tumor immune milieu [23].

### Aptamer-siRNA-mediated metabolic and apoptotic reprogramming disrupts tumor-infiltrating Treg cells

We previously identified that tumor-infiltrating Treg cells migration is critically dependment on glycolysis, and that pharmacological inhibition of glycolysis (e.g., with 2-deoxy-D-glucose, 2DG) impairs their infiltration within tumors. Given that mTORC1 is a key upstream regulator of cellular metabolic programs, notably glycolysis, we hypothesized that aptamer-siRNA may modulate Treg cell trafficking via altering their metabolic reprogramming [24].

**Fig. 5:**
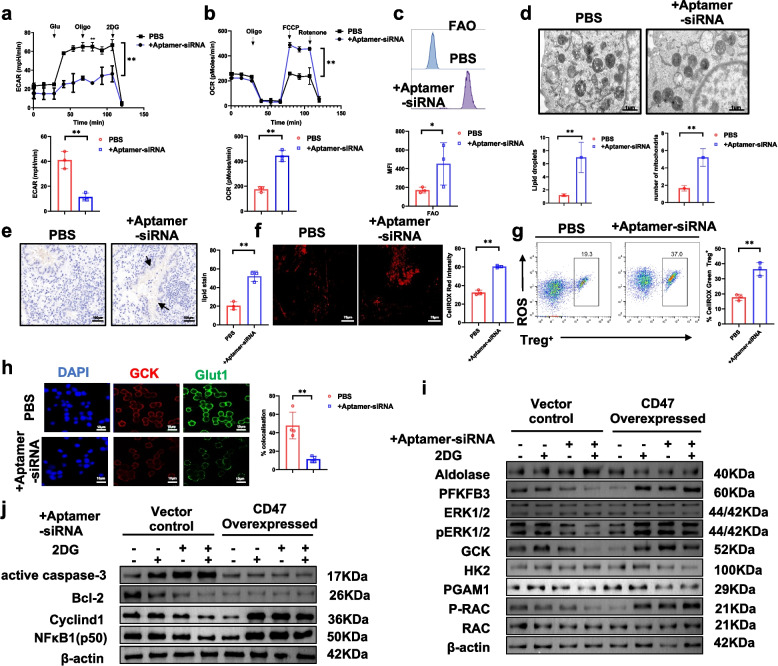
Aptamer-siRNA drives metabolic and mitochondrial remodeling and alters apoptosis and proliferation signaling in tumor-infiltrating Treg cells. **a** ECAR assays showing a significant decrease in glycolytic activity in aptamer-siRNA-treated Treg cells. **b** OCR assays indicating increased mitochondrial respiration following aptamer-siRNA treatment. **c** FAO levels were significantly elevated in the aptamer-siRNA group, as assessed by flow cytometry using a fluorescent FAO-specific probe FAOBlue. **d** Transmission electron microscopy (TEM) images revealing increased numbers of mitochondria and lipid droplets in aptamer-siRNA-treated cells. Scale bar, 2 μm; 500 cells were analysed per sample. **e** Lipid staining demonstrating enhanced intracellular lipid droplet accumulation after aptamer-siRNA treatment. Scale bar, 100 μm. **f** Confocal microscopy of ROS fluorescence (MitoSOX Red) showing increased mitochondrial ROS levels in the aptamer-siRNA group. Scale bar, 75 μm; 500 cells were analysed per sample. **g** Flow cytometry analysis using MitoSOX Green demonstrated that aptamer-siRNA treatment increased the proportion of Treg + ROS +. **h** Confocal images showed a decrease in glucose metabolism in the aptamer-siRNA-treated group compared to the control group (PBS). For quantification, 500 cells per coverslip were analysed, and the bar graph illustrates the percentage of cells displaying Glut1 and GCK localization in treated Treg cells, Scale bar, 10 μm. **i** Western blot analysis of glycolysis-related proteins in vector control and CD47-overexpressing groups following treatment with aptamer-siRNA or 2-deoxy- D-glucose (2-DG). **j** Western blot analysis of apoptosis and proliferation markers (Cyclin D1, NF-κB, active Caspase-3, and Bcl-2) under the indicated conditions. Data are presented as mean ± s.d. from three independent experiments. Statistical significance was assessed by two-tailed Student’s t-test.*P < 0.05, **P < 0.01, ***P < 0.001, ****P < 0.0001

Our results showed that aptamer-siRNA treatment significantly reduced the ECAR, a surrogate measure of glycolytic flux and lactate production, reflecting suppressed glycolytic activity in tumor-associated Treg cells (Fig. 5a). In contrast, OCR measurements indicated an increase in mitochondrial respiration, consistent with a potential metabolic adjustment in response to glycolytic suppression (Fig. 5b). In line with this shift, aptamer-siRNA markedly increased FAO activity (Fig. 5c) and promoted intracellular lipid droplet accumulation (Fig. 5d-e). Notably, mitochondrial oxidative stress (ROS) was substantially elevated, as evidenced by intensified MitoSOX Red fluorescence in confocal microscopy (Fig. 5f) and a higher proportion of ROS⁺ CD4⁺CD25⁺ Tregs identified by flow cytometry using CellROX Green staining (Fig. 5g). In addition, confocal microscopy revealed downregulation of Glut1 and glucokinase (GCK), both of which are involved in glucose uptake and metabolism, further indicating a potential impairment in glycolytic capacity following conjugate treatment (Fig. 5h).

To clarify the molecular mechanisms involved, we generated tumor-infiltrating Treg cells with stable CD47 overexpression via lentiviral transduction and analyzed their protein expression by Western blot. In control Treg cells, aptamer-siRNA treatment led to marked downregulation of glycolysis-associated proteins (PFKFB3, p-ERK1/2, p-Rac, GCK), and these effects were enhanced by 2DG, confirming glycolysis dependence. In contrast, CD47 overexpression reversed the inhibitory effects of aptamer-siRNA, suggesting compensatory activation of glycolytic (Fig. 5i). In addition, aptamer-siRNA reduced Cyclin D1, Bcl-2, and NF-κB levels while increasing active caspase-3, indicating impaired survival and increased apoptosis. These changes were diminished in CD47-overexpressing Tregs, highlighting the importance of CD47 in maintaining Treg fitness (Fig. 5j). Subsequently, analysis of the PD-L1 pathway revealed that aptamer-siRNA treatment led to upregulation of pro-apoptotic markers and downregulation of anti-apoptotic markers in Treg cells. These effects were further amplified by PD-L1 knockdown and attenuated by PD-L1 overexpression, demonstrating that PD-L1 plays a critical role in maintaining Treg cell survival within the TME. Additionally, inhibition of PD-L1 signaling was associated with reduced levels of glycolysis-related proteins, suggesting that PD-L1 may also support Treg cell metabolic activity (Fig. S4a-h).

Given the well-established interplay among TCR signaling, glycolytic metabolism, and the ERK/mTOR pathways, we systematically assessed Treg activation, metabolic reprogramming, and migratory capacity. Specifically, we examined key TCR signaling markers (pERK1/2, pZAP70), downstream mTOR effectors (mTOR, pS6K), and the surface activation marker CD69. Glycolytic activity was quantified by measuring 2-NBDG uptake and GLUT1 expression, while migratory capacity was evaluated using chemotaxis assays. Our results demonstrate that blockade of CD47 resulted in a pronounced reduction in glycolytic activity and a significant impairment of migratory capacity (Fig. S4 i-j, p). Notably, TCR activation did not markedly enhance Treg migration following CD47 knockdown compared to the unstimulated group, suggesting the critical role of CD47 in regulating Treg migration. In contrast, inhibition of the PD-L1 axis primarily attenuated Treg activation and glycolytic activity, while exerting limited impact on their migration (Fig. S4k-o). Investigation of the concurrent blockade of both CD47 and PD-L1 pathways revealed a synergistic effect, with markedly greater impairment of Treg function and migration than observed with single pathway inhibition. Collectively, these findings indicate that the observed effects are not independently modulated, but instead reflect a coordinated disruption across multiple functional axes, simultaneously impacting both Treg activation/function and migration/chemotaxis pathways.

### Metabolite pathway enrichment analysis reveals proline metabolic pathway modulation in liver cancer treatment with aptamer-siRNA

**Fig. 6:**
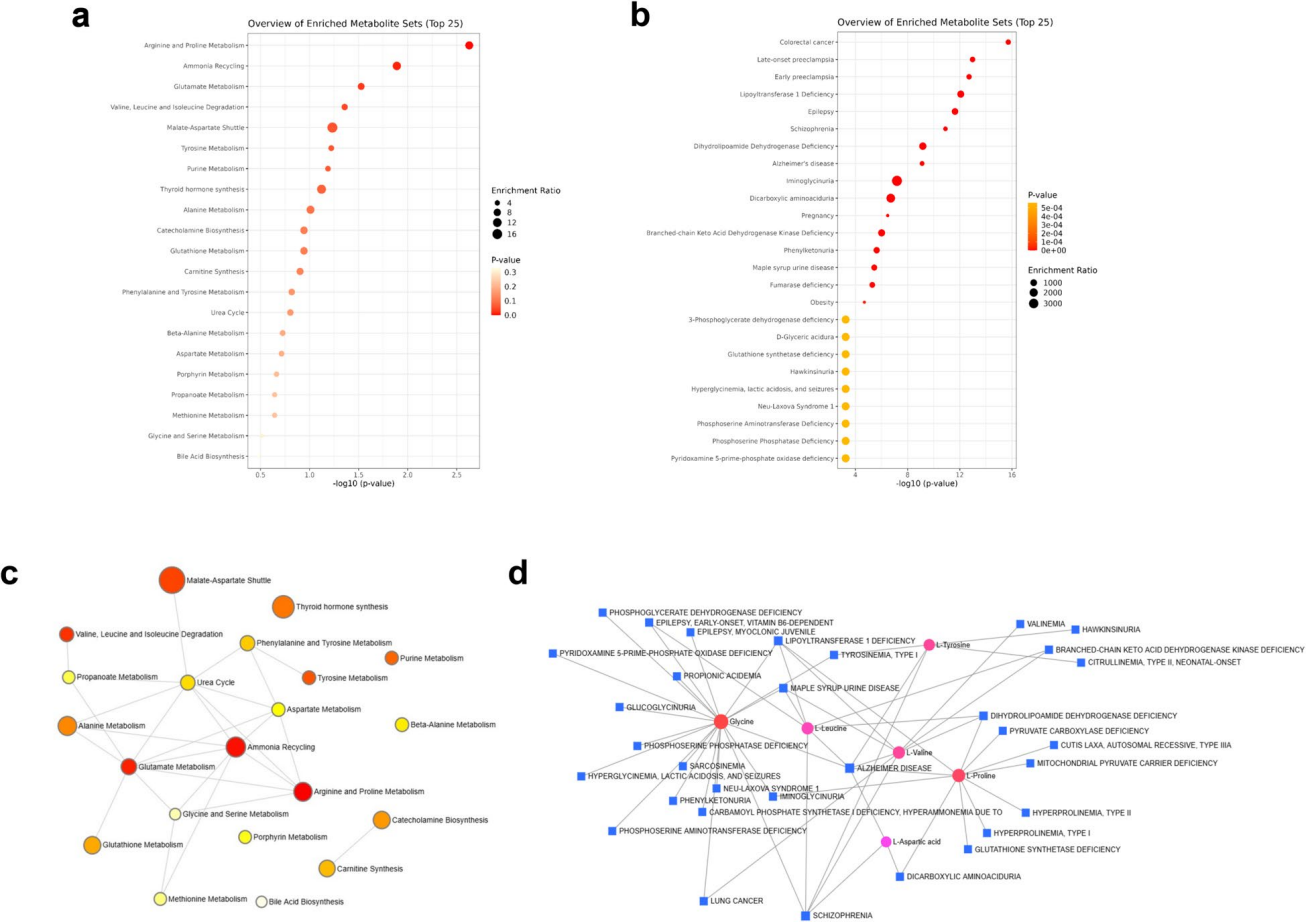
Aptamer-siRNA chimera alters proline metabolic pathways and immune regulation in the liver tumor microenvironment. **a-b** KEGG enrichment analysis revealing significant enrichment in amino acid metabolism (**a**) as well as metabolic-related disease (**b**), displaying the top 25 metabolite sets. **c** Network analysis illustrating the relationships among amino acid metabolism pathways, corresponding to the data in panel A. **d** Disease Signatures Enrichment Analysis based on amino acid metabolism (aspartic acid, glycine, leucine, tyrosine, valine, and proline) identifies proline as a central metabolite

To investigate the metabolic consequences of aptamer-siRNA therapy in tumor-infiltrating Treg cells, we performed targeted serum metabolomic profiling in mice bearing liver tumors and treated with saline, anti-PD-L1 aptamer, CD47 siRNA, or the aptamer-siRNA chimera. The results showed that treatment with the aptamer-siRNA chimera induced pronounced alterations in the levels of aspartic acid, glycine, leucine, tyrosine, valine, lysine, and proline (Fig. S5). KEGG pathway and network analyses identified significant modulation of arginine and proline metabolism, as well as pathways involved in ammonia recycling, malate-aspartate shuttle and valine, leucine and isoleucine degradation (Fig. 6a, c). Disease signature enrichment analysis demonstrated that these metabolic changes overlap with several metabolic disorders, including lipoyltransferase 1 deficiency, dihydrolipoamide dehydrogenase deficiency, iminoglycinuria, and dicarboxylic aminoaciduria, highlighting the clinical relevance of these pathways (Fig. 6b). Notably, proline emerged as a central metabolite in disease signature enrichment, with the spectrum of associated diseases closely recapitulating the enrichment pattern observed in panel B (Fig. 6d). These results suggest that aptamer-siRNA treatment substantially reprograms amino acid metabolic networks in tumor-infiltrating Treg cells, with a particular impact on proline metabolism. Given that proline metabolism is essential for maintaining redox balance, supporting energy production, and facilitating extracellular matrix synthesis processes implicated in both tumor progression and immune regulation, the observed reduction in proline levels may reflect a shift away from immunosuppressive metabolic programs within the TME [25–27].

## Discussion

Tumor-infiltrating Treg cells play a critical role in suppressing antitumor immune responses and are often associated with poor prognosis across various cancer types [28, 29]. Treg cells suppress effector T cell functions through multiple immunosuppressive mechanisms. Notably, even apoptotic intratumoral Treg cells have been shown to attenuate the efficacy of checkpoint blockade immunotherapy by producing immunosuppressive adenosine [30]. Recent preclinical models and clinical trials have explored therapeutic targeting of CD47, which has shown promising results for B cell lymphoma when combined with tumor-targeting antibodies [5]. However, addressing the CD47 antigen sink and limited therapeutic activity observed in solid tumors remains challenging.

We rationally designed an anti-PD-L1 × CD47 aptamer-siRNA chimera to simultaneously block two distinct but complementary immune evasion pathways in tumor-infiltrating Treg cells. PD-L1 suppresses cytotoxic T cell activity via the PD-1 signaling, while CD47 engages SIRPα on macrophages to inhibit phagocytosis through the “don’t eat me” signal. Treg cells within the TME often co-express both PD-L1 and CD47, allowing them to evade both adaptive and innate immune responses [31]. By targeting PD-L1 for selective delivery and silencing CD47 via siRNA, this chimera enables dual checkpoint blockade and electively accumulates in tumor-infiltrating Treg cells. This strategy not only promotes functional depletion of intratumoral Tregs, but also minimizes off-target effects in peripheral cells such as spleen Tregs and RBCs. The synergistic design is expected to enhance therapeutic precision while reducing systemic toxicity, offering a more effective and translatable approach than conventional antibody combinations.

Recent studies have focused on modifying the SIRP/CD47 pathway to influence innate immune cells, particularly macrophages, for cancer treatment. For example, Zhang et al. improved anti-CTLA-4 responses against colorectal cancer by inhibiting the SIRP/CD47 pathway [3]. Clinically, preferential depletion of Tregs in the TME may be more desirable than systemic blockade of CTLA-4 function. While CTLA-4 remains a classical and widely used target for Treg depletion (e.g., ipilimumab), our study instead utilizes PD-L1 for selective delivery. PD-L1 is frequently upregulated in tumor-infiltrating Treg cells and allows for dual functional roles both as a target for checkpoint inhibition and as a selective delivery handle. Compared to CTLA-4 blockade, which can lead to widespread systemic Treg depletion and immune-related adverse events, PD-L1 targeting may offer improved intratumoral specificity with reduced peripheral toxicity. This selective delivery is achieved through aptamer-guided recognition of PD-L1⁺ cells within the TME, ensuring effective CD47 silencing with limited interaction with CD47⁺ red blood cells or splenic Tregs.

Mechanistically, our data illuminate the central role of selective Treg depletion and exhaustion in shaping the antitumor immune landscape following PD-L1 × CD47 aptamer-siRNA chimera treatment, while sparing the overall CD4⁺ T cell population and non-Treg subsets such as Th1 cells. This selective effect reflects targeted exhaustion and depletion rather than global CD4⁺ T cell suppression, thus minimizing the risk of broad immunodeficiency. Intriguingly, the attenuation of Treg-mediated immune suppression coincided with a significant expansion of both Th17 and CD8⁺ T cells within the TME. The augmented frequency and absolute number of CD8⁺ T cells, together with upregulated expression of functional markers such as IFN-γ, granzyme B, and CD69, indicating heightened cytotoxic potential. Relief of inhibitory constraints imposed by Tregs fosters CD8⁺ T cell proliferation, activation, and effector differentiation, collectively contributing to the establishment of a pro-inflammatory and cytolytic TME conducive to robust antitumor immunity [32]. Notably, this immunological remodeling is not accompanied by alterations in macrophage number or polarization, but is instead characterized by elevated phagocytic activity, further supporting the specificity of the intervention toward reversing Treg-mediated immune suppression.

Beyond immunophenotypic alterations, we provide compensatory evidence that the PD-L1 × CD47 aptamer-siRNA chimera achieves selective intratumoral Treg depletion accompanied by orchestrating multifaceted disruptions in Treg functional fitness. Our results demonstrate that inhibition of PD-L1 and CD47 signals profoundly impairs glycolytic metabolism in Treg cells, as reflected by reduced ECAR and upregulation of FAO, ultimately leading to mitochondrial stress and apoptosis. This metabolic shift is further accompanied by decreased expression of glycolysis-associated enzymes such as GLUT1 and GCK, highlighting the critical role of glycolysis in the metabolic plasticity of tumor-infiltrating Treg cells, enabling their adaptation and sustained immunosuppressive function [33]. This observation also supports the notion that efficient Treg trafficking relies on glycolytic activity [34].

Furthermore, by converging on multiple regulatory axes, the chimera impairs key intracellular networks governing Treg activity, survival, and migration. Specifically, blockade of the CD47 axis destabilizes processes fundamental to Treg motility, metabolic homeostasis, and persistence, thereby undermining their capacity to accumulate within the tumor niche. Complementarily, interference with PD-L1 signaling exerts a significant inhibitory effect on Treg activation, as reflected in the downregulation of canonical activation pathway and effector function, which is consistent with previous studies [35]. This integrated strategy results in a state of functional exhaustion, characterized by blunted antigen-driven responsiveness and heightened vulnerability to apoptotic signals [36].

We also interrogated the broader metabolic consequences of aptamer-siRNA therapy by assessing serum amino acid levels. Treatment led to significant alterations in the metabolic profile, with KEGG enrichment analysis highlighting proline metabolism. Previous studies have shown that proline metabolic dysregulation closely correlates with tumor cell proliferation, invasion, and modulation of the immune microenvironment [37]. The pronounced reduction of proline observed in our study suggests that aptamer-siRNA therapy may remodel Treg cell amino acid metabolic networks, thereby potentially influencing their immunosuppressive function and promoting antitumor immunity.

From a translational perspective, clinical efficacy of PD-1/PD-L1 antibodies in solid tumors is frequently constrained by limited tumor penetration, the occurrence of immune-related adverse events associated with systemic immune activation, and substantial production costs [38]. In contrast, aptamer-based therapeutics offer rapid, cost-effective synthesis and superior tissue penetration due to their small size, enabling highly specific targeting within the TME [39]. Such selectivity is particularly critical in solid tumors to minimize systemic toxicity and off-target immune activation. In our study, aptamer-siRNA chimeras achieved dual checkpoint inhibition with precise cellular specificity, resulting in efficient Treg depletion and robust CD8⁺ T cell activation while sparing normal tissues.

**Fig. 7:**
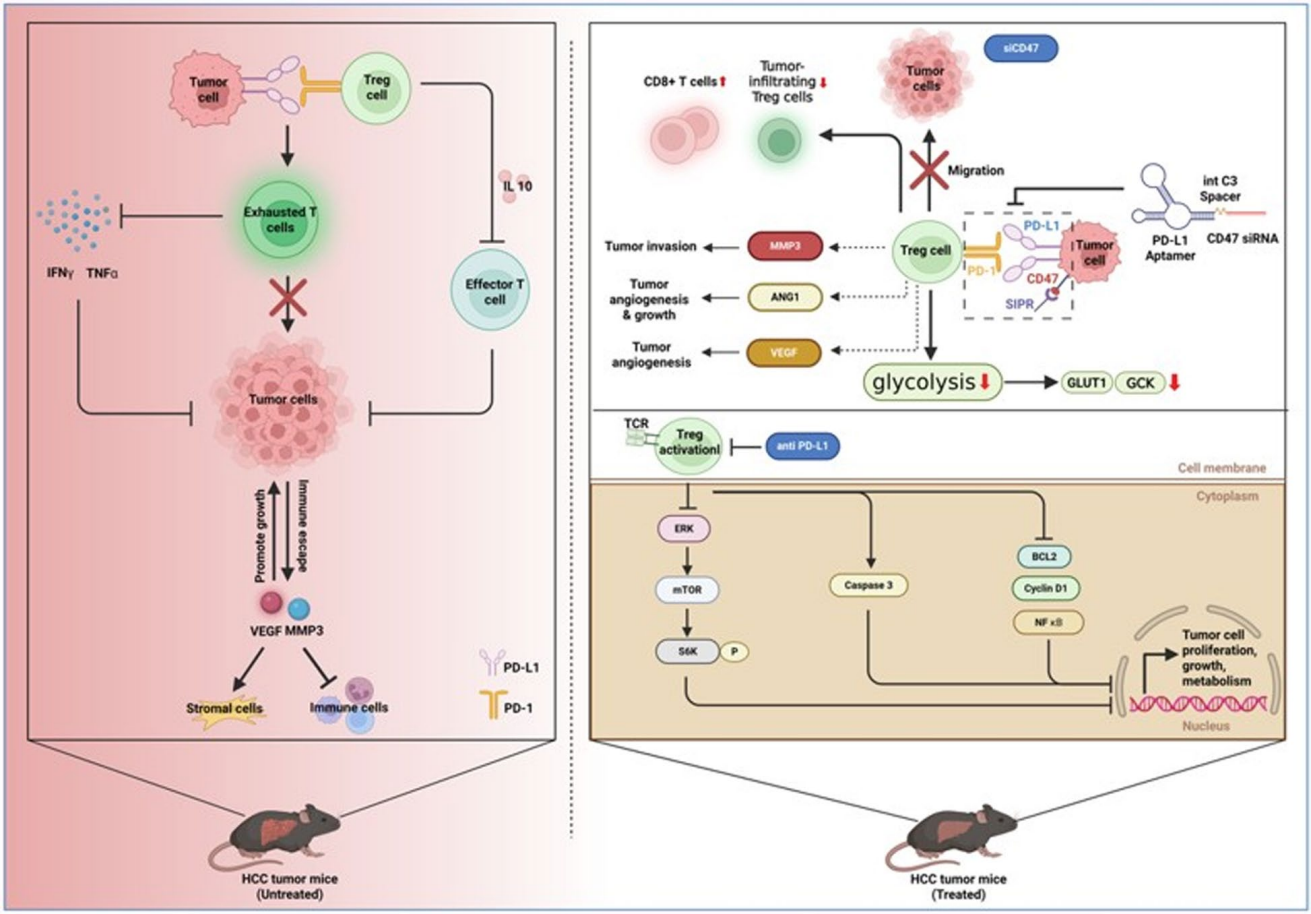
Interactions between tumor cells and immune cells and the effects of therapeutic intervention in mouse models of HCC. The PD-L1 × CD47 aptamer-siRNA chimera simultaneously blocks the PD-L1 immune checkpoint and silences CD47 expression in tumor-infiltrating Treg cells. This dual targeting disrupts Treg immunosuppressive function and metabolic activity, leading to their depletion. Consequently, cytotoxic CD8⁺ T cell infiltration and function are restored, resulting in suppressed tumor growth and angiogenesis within the TME

In conclusion, our study demonstrates that the PD-L1 × CD47 aptamer-siRNA chimera represents a novel and effective strategy for selectively depleting tumor-infiltrating Treg cells and enhancing antitumor immunity (Fig. 7). While these findings highlight the therapeutic promise of this approach, we acknowledge certain limitations, including its evaluation in a single murine HCC model and the need to further elucidate the upstream signaling mechanisms underlying the observed metabolic reprogramming. Future studies incorporating additional tumor models and mechanistic investigations will be valuable for advancing this RNA-based immunotherapeutic platform toward clinical translation.

## Methods

### Mice

All the animal experiments were reviewed and approved by the ethics committee of Hong Kong Baptist University (REC/23–24/0032). 10 × 10⁶ Hepa1-6 murine cells were subcutaneously injected into the rear flank of C57BL/6J mice. Starting on day 7 post-inoculation, mice received intravenous injections of anti-PD-L1 × CD47 siRNA at a dose of 2 mg/kg every 3 days, continuing until day 29. Control mice were treated with an equivalent volume of PBS. Throughout the study, tumor burden was carefully monitored, and all mice were euthanized before any tumor reached 15 mm in diameter, 2000 mm3 (calculated as length × width2/2) in volume, or exceeded 10% of body weight, in accordance with international ethical standards.

### Cell lines and reagents

Treg cells were isolated from mouse tissues by immunomagnetic bead separation and detected by flow cytometry. Briefly, spleens and tumor tissues were harvested from C57BL/6J mice, and single-cell suspensions were prepared by mechanical dissociation and filtration through a 70 μm cell strainer. RBCs were lysed using ACK lysis buffer. Treg cells were separated using a mouse CD4^+^CD25^+^ regulatory T cell isolation kit (Miltenyi, Germany) according to the manufacturer’s protocols. The purity of Treg cells was verified by flow cytometry. To verify the purity of Treg cells, we used antibodies to label CD4, CD25, and Foxp3 to distinguish Treg cells (CD4^+^CD25^+^Foxp3^+^). Then, double-positive (CD4^+^CD25^+^) and Foxp3^+^ cells were screened by flow cytometry and their proportion of total cells was calculated, and the purity reached more than 90%.

### Synthesis and conjugation of aptamer-siRNA

We designed a loss-gain cell-SELEX strategy to select aptamers against PD-L1 protein which could specifically recognize, tightly bind to, efficiently inhibit PD-1/PD-L1 interaction. The anti-PDL1 aptamer and CD47 siRNA were synthesized and purified by Genewiz. An internal C3 spacer was used to link the aptamer and siRNA to form a Cy5-labeled conjugate (5’-Cy5-dAdCdCdGdAdCdCdGdTdGdCdTdGdGdAdCdTdCdAdTdCdTdCdGdCdTdTdTdTdTdTdCdAdCdGdGdTdCdCdAdCdAdCdTdAdCdTdAdTdGdAdGdCdGdAdGdCdCdTdGdGdCdG-iSpC3-rGrGrArArUrGrArCrCrUrCrUrUrUrCrArCrCrA-3′5’-rUrGrGrUrGrArArArGrArGrGrUrCrArUrUrCrC-3’) [20, 40].

### Enzyme-Linked Oligonucleotide Assay (ELONA)

Human recombinant PD-L1 protein (160 ng) was coated into a 96-well microtiter plate in 100μL of PBS and incubated at 4 °C overnight. The plate was then blocked using blocking buffer (PBS, 0.1% Tween 20 and 1% BSA) for 1h at room temperature and washed with washing buffer (PBS, 1 mM MgCl_2_, 0.1% Tween 20 and 0.1% BSA) for 4 times. The aptamer/aptamer-siRNA conjugate was denatured at 95 °C for 10 min and rapidly cooled on ice for 10 min to ensure proper refolding into its functional three-dimensional structure for optimal binding activity. Appropriate concentrations of biotinylated aptamer/aptamer-siRNA were added to each well (100μL) and incubated for 45 min at room temperature with continuous gentle shaking. A nonlinear curve fitting model Hyperbl was used to plot the binding curve. The equation of the Hyperbl model is y = P1*X/(P2 + X), and P2 is the Kd value [41]. The Hyperbl model was selected based on the assumption of a simple 1:1 binding interaction between the aptamer and PD-L1, which is appropriate when the binding curve lacks a sigmoidal shape and the data do not exhibit saturation behavior typical of cooperative systems.

### Quantification of cellular uptake

Quantification of the Cy5-labeled aptamer/siRNA/aptamer-siRNA uptake into cells was performed by measuring fluorescence intensity using a microplate fluorescence reader with an excitation wavelength of 650nm and an emission wavelength of 675nm. Treg cells were seeded in a 12 well plate at a density of 2 × 10^5^ per well and cultured overnight. Cells were then treated with 250nM of 5’-Cy5-labeled aptamer, siRNA, aptamer-siRNA or vehicle, and incubated at 37 °C for 3h.

### In vitro gene knockdown efficiency

To evaluate knockdown in vitro, Treg cells were treated with aptamer, siRNA, aptamer-siRNA, or vehicle in serum-free medium for 8 h. After switching to complete medium (10% FBS) for 48 h, CD47 mRNA levels were measured using real-time qPCR [42].

### Cellular internalization assay by confocal imaging

For confocal imaging, Treg cells were plated overnight in glass-bottom dishes at 5 × 10^4^ cells/well. They were then co-incubated for 3 h at 37 °C with 250 nM of the Cy5-labeled aptamer-siRNA chimera and Alexa Fluor 488-tagged endocytic tracers (dextran, transferrin, or CTX-B). Subsequently, cells were washed with PBS, fixed in 4% PFA for 10 min, stained with DAPI, and washed again prior to imaging [43]. Confocal images were acquired using a Zeiss LSM510 or Axioplan2 microscope equipped with a 60 × objective, and processed with LAS X software (Leica Microsystems) without further modification.

### In vitro tumor infiltrating Treg migration and chemotaxis assays

Tumor-infiltrating CD4⁺CD25⁺Foxp3⁺ Treg cells treated with PD-L1 aptamer, CD47 siRNA, or aptamer-siRNA chimera were resuspended in RPMI 1640 supplemented with 2% FBS and seeded into the upper chambers of Transwell inserts (5 μm pore size, Corning) at a density of 1 × 10^5^ cells per well. The lower chambers were filled with medium containing recombinant mouse IP10 (CXCL10;100 ng/ml). After incubation for 24 h at 37 °C in a 5% CO₂ incubator, cells that had migrated to the lower chamber were collected and quantified by flow cytometry using counting beads.

### RBCs binding assay

Peripheral blood was collected from mice into heparinized tubes and centrifuged at 500 × g for 5 min at 4 °C to isolate RBCs. The harvested RBCs were washed three times with cold PBS and then resuspended at a final density of 1 × 10^6^ cells/mL. For binding assays, 250 nM Cy5-labeled PD-L1 aptamer, CD47 siRNA, or aptamer-siRNA conjugate were added to the RBC suspension (200 μL total volume) and incubated at room temperature for 30 min in the dark. Following incubation, cells were washed twice with PBS to remove unbound oligonucleotides, and mean fluorescence intensity (MFI) was quantified using flow cytometry (BD FACS Verse™ flow cytometer).

### Measurement of extracellular acidification rates (ECAR) and oxygen consumption rates (OCR)

Treg cell bioenergetics were assessed by measuring ECAR and OCR in real time on a Seahorse XF analyzer. Prior to assay, cells were incubated for 1 h in the manufacturer’s unbuffered XF medium (Cat#102,365–100) and plated at 6 × 10^5^ cells per well in an XF24 plate. To profile metabolic pathways, the following compounds were injected: oligomycin, FCCP, antimycin A, and rotenone (each 1 μM), followed by D-glucose (10 mM) and 2-DG (50 mM; all from Seahorse, Cat#103,020–100, 103,015–100). Measurements were taken under standard cycling conditions (2 min mix, 2 min wait, 4–5 min measure) and analyzed with Wave v2.4.1 software.

### Electron microscopy of resin-embedded cells

Treg cell samples were collected by filtration, frozen in a Bal-Tec HPF010 high-pressure freezer, and then transferred to a Leica AFS unit for freeze substitution. This process involved storage at −85°C for 60 h and a slow 18 h warming phase to 0°C. Finally, the prepared sections were stained with uranyl acetate and lead citrate and examined using a Hitachi H7650 TEM at an accelerating voltage of 80 kV.

### Liquid chromatography-mass spectrometry (LC–MS) identification

The amino acids were assayed utilizing a Waters ACQUITY UPLC system coupled to a mass spectrometer. Chromatographic separation was performed on a Waters® BEH C18 column (1.7 μm, 2.1 mm × 100 mm). Mobile phase A consisted of water with 0.1% formic acid, and mobile phase B was methanol with 0.1% formic acid. A linear gradient elution was employed (initially 5% B for 0.5 min, ramped to 100% B over 5 min) at a flow rate of 0.2 mL/min. Prior to analysis, samples were derivatized using AccQ-Tag reagent kit (Waters, Milford, MA, USA) following the manufacturer’s instructions.

### Flow cytometry analysis

For surface marker staining, cells were resuspended in 100µL of staining buffer (PBS with 2%FBS) and incubated with fluorescence-conjugated antibodies at the appropriate dilution as recommended by the manufacturer. Cells were then stained with anti-CD4 (mouse) (Sigma-Aldrich, MABF157B); anti-CD47 (mouse) (BioLegend, 127,501); anti-PD-L1 (mouse) (BioLegend, 124,301); CD206 (mouse) (Sigma-Aldrich, ZRB1440); anti-CD80 (B7-1) (mouse) (Sigma-Aldrich, MABF455); anti-CD11b (mouse) (BioLegend, 101,201); anti-CD45 (mouse) (BioLegend, 103,101); anti-F4/80 (mouse) (Abcam, ab6640); anti-CD3 (mouse) (BioLegend, 100,205), anti-CD8 (BioLegend, 100,712), anti-Ly6C (mouse) (BioLegend, 128,023), anti-11C(mouse) (BioLegend, 117,305), anti-MHC II (mouse) (BioLegend, 107,605). anti-IL-17A (mouse) (BioLegend, 506,901); monoclonal anti-FOXP3-PE antibody (Sigma-Aldrich, SAB4700611); anti-Vascular Endothelial Growth Factor Receptor 2 antibody (Sigma-Aldrich, V1014); MMP3 polyclonal antibody (Invitrogen, #PA5-119,639); Angiopoietin 1 polyclonal antibody (Invitrogen, #PA5-142,603) for 30 min at 4 °C in the dark.

### Western Blot

Tumor-infiltrating CD4⁺CD25⁺ Treg cells were isolated from Hepa1-6 tumor-bearing mice using immunomagnetic bead separation (Miltenyi Biotec). Treg cells were lysed immediately after isolation without ex vivo restimulation to preserve their native activation status. Western blot using the following primary antibodies: anti-PFKFB3 (Sigma-Aldrich, SAB4301856); anti-Phospho-Erk1/2-T202/Y204 antibody (Sigma-Aldrich, SAB5701896); pRAC polyclonal antibody (Invitrogen, PA5-117,342); RAC1 polyclonal antibody (Invitrogen, PA1-091); monoclonal anti-HK2 (Sigma-Aldrich, SAB1402221); anti-GCK antibody (Abcam, ab250726); anti-Aldolase antibody (Abcam, ab245469); anti-PGAM1 antibody (Sigma-Aldrich, SAB2500780); anti-beta-Actin antibody (Sigma-Aldrich, SAB5600204); anti-Cyclin D1 antibody (Sigma-Aldrich, SAB5701174); monoclonal anti-NF-κB antibody (Sigma-Aldrich, N8523); anti-Caspase 3 antibody (Sigma-Aldrich, C8487); anti-Bcl-2 antibody (Sigma-Aldrich, MABC573); VEGF (CST, #2463); ANG1(Abcam, ab94684); MMP3(Abcam, ab52915). After incubation with HRP-conjugated secondary antibodies, protein bands were visualized using ECL substrate (Thermo Fisher) and imaged on a ChemiDoc imaging system (Bio-Rad).

### Immunohistochemistry

Immunohistochemistry was performed as follows: after dewaxing and rehydration, endogenous peroxidase was eliminated by hydrogen peroxide (3%). The slides were then incubated at 4 °C overnight with primary antibodies: anti-IL-17A antibody (Sigma-Aldrich, ZRB2409); and anti-Foxp3(Abcam, ab20034); anti-Ki67 antibody (Abcam, ab197547). Quantification of staining was performed on 10 random 40 × fields. The percentage of positive tumor cells per field determined the score: 0 (negative), 1 (< 25%), 2 (25–50%), or 3 (> 50%). The final score was derived from the summation of all individual field scores.

### Stimulation of Treg cells with anti-CD3 plus anti-CD28 mAb

Tumor-infiltrating Treg cells were isolated from murine liver tumor tissues using the Mouse CD4 + CD25 + Regulatory T Cell Isolation Kit (Miltenyi Biotec), according to the manufacturer’s instructions. The purified cells were plated at 1.5 × 10^6^ cells/well in 24-well plates pre-coated with anti-mouse CD3ε antibody (clone 145-2C11, 5 μg/mL), and supplemented with soluble anti-mouse CD28 antibody (clone 37.51, 2 μg/mL) in the presence of recombinant mouse IL-2 (Peprotech, Cat# 212–12). The culture medium consisted of RPMI-1640 (Gibco), 10% FBS, 1% Penicillin–Streptomycin, and 50 μM 2-mercaptoethanol (Sigma-Aldrich). Cells were incubated at 37 °C, 5% CO₂ for 48 h prior to subsequent analysis.

### STRING database analysis and pathway enrichment

A list of differentially expressed genes (DEGs) was generated based on PD-L1/SIRP target genes. Protein–protein interaction (PPI) network analysis was conducted using the STRING database (version 11.5; https://string-db.org/). The resulting PPI network was visualized to explore possible functional interactions among the DEGs. Functional enrichment analysis, including annotation of Gene Ontology (GO) terms and Kyoto Encyclopedia of Genes and Genomes (KEGG) pathways, was conducted within the STRING platform using its integrated enrichment algorithms. Biological processes and pathways with a false discovery rate (FDR) < 0.05 were considered statistically significant.

### Statistical analysis

All data analysis was carried out using GraphPad Prism 10 and SPSS 16.0. For the majority of in vitro and animal experiments, one-way ANOVA or an unpaired two-tailed Student’s t-test were applied to generate P values, with post hoc tests used to determine specific differences between groups. Data were presented as mean ± SD of independent experiments triplicates. A P-value of less than 0.05 was considered statistically significant.

## Supplementary Information

Supplementary Material 1: Fig. S1 Pathway enrichment analysis of PD-L1- and CD47-associated genes.a KEGG pathway enrichment of genes associated with PD-L1 and CD47 (IL2, IFNG, TNF, TBX21, EOMES, CTLA4, LAG3, BCL-2, BAX, GLUT1, HK2, IL-6, IL-12, IL-10. TGF-beta, Rac1, and mTOR1). bEnrichment analysis using the Elsevier Pathway Collection, highlighting the involvement of target genes in cancer immune evasion mechanisms, including IDO1-mediated immune escape and effector T cell inactivation. c-d Comprehensive pathway and network analyses were performed to elucidate that the targeting genes are closely associated with immune response pathways and metabolic processes. Fig. S2.Aptamer-siRNA promotes apoptosis and inhibits proliferation in subcutaneous liver tumors.a Quantitative analysis of angiogenesis-related markers (VEGF, MMP-3, and ANG-1) by flow cytometry in tumours treated or not treated with aptamer-siRNA. b Ki67 staining to assess cell proliferation in tumour sections. Scale bar, 100µm. Data are presented as mean ± s.d. (n = 3 per group). c Representative TUNEL staining to analyses the proportion of apoptotic cells. Scale bar, 100µm. Statistical analysis was performed using one-way ANOVA with Tukey’s post hoc test.*P< 0.05, **P< 0.01, ***P < 0.001*.*Fig. S3.Aptamer-siRNA chimera treatment alters the number of Treg cells in tumours without affecting macrophage polarization. a Absolute numbers of total CD4⁺ T cells, Treg cells (FoxP3⁺CD4⁺), Th1 cells (IFN-γ⁺CD4⁺), and Th17 cells (IL-17A⁺CD4⁺) were determined by flow cytometry in healthy spleen, PBS-treated tumors, and chimera-treated tumors. b Flow cytometric analysis of tumour‑associated macrophage polarization showing no significant changes following aptamer-siRNA treatment. c-d Flow cytometry analysis of intratumoral CD3⁺CD8⁺ T cells in healthy, PBS-treated, and chimera-treated tumors, presenting both the proportion of CD8⁺ T cells among CD45⁺ cells and the absolute number per milligram of tumor tissue. e-g Quantification of the proportion of (e) CD69⁺, (f) IFN-γ⁺, and (g) Granzyme B⁺ cells among total CD8⁺ T cells in tumor tissues. h-i Analysis of the frequency and absolute number of intratumoral monocytes following PD-L1×CD47 aptamer-siRNA chimera treatment. j-k Analysis of the frequency and absolute number of intratumoral dendritic cells (DCs) following PD-L1×CD47 aptamer-siRNA chimera treatment. Data are presented as mean ± s.d. (n=3 per group). Statistical significance was assessed using one-way ANOVA followed by Tukey’s multiple comparisons test or two-tailed Student’s t-test. ns, not significant; *P < 0.05，**P < 0.01*.*Fig. S4.Effects of PD-L1 modulation and metabolic intervention on Treg cell function. a-h Quantitative PCR analysis of PFKFB3, GCK, BCL2, CYCLIN D1 and NFKB1 mRNA levels, and ELISA measurement of p-ERK1/2, p-RAC and CASPASE3 protein expression in Treg cells across all groups. i–j Metabolic activity of tumor-infiltrating Tregs, assessed by (i) 2-NBDG uptake, and (j) GLUT1 expression, following aptamer-siCD47 treatment with or without TCR stimulation (αCD3/CD28). k-o Levels of (k) pERK1/2, (l) pZAP70, (m) pS6K, (n) mTOR, and (o) CD69 in tumor-infiltrating Tregs were quantified by flow cytometry or qPCR following aptamer-siCD47 treatment with or without TCR stimulation (αCD3/CD28). p Number of migrated Treg cells upon TCR stimulation with αCD3/CD28.Data are mean ± s.d. (n = 3 per group). Statistical analysis was performed using one-way ANOVA with Tukey’s post hoc test. *P < 0.05, **P < 0.01, ***P <0.001, ****P < 0.0001. Fig. S5. Amino acid metabolic enrichment analysis. Amino‑acid levels in tumour‑infiltrating regulatory T cells isolated from liver tumours were measured following treatment with saline, anti‑PD‑L1 aptamer, CD47 siRNA or the aptamer-siRNA chimera. Data are presented as mean ± s.d. (n = 3 per group). Statistical significance was determined by Student’s t-test: *P< 0.05, **P < 0.01, ***P <0.001, ****P < 0.0001.

## Data Availability

All data generated or analyzed during this study are included in this published article and its supplementary information files. Further details are available from the corresponding author upon reasonable request.
