# Supplementary material for: Targeting tumor-infiltrating regulatory T cells: combining CD47 and PD-L1 inhibition via a novel aptamer-siRNA chimera
Source: Mol Biomed. 2025 Dec 17;6:140. doi: 10.1186/s43556-025-00392-2 (PMC12708450; doi:10.1186/s43556-025-00392-2)
Supplement: Supplementary file 1 — Supplementary Material 1: Fig. S1 Pathway enrichment analysis of PD-L1- and CD47-associated genes.a KEGG pathway enrichment of genes associated with PD-L1 and CD47 (IL2, IFNG, TNF, TBX21, EOMES, CTLA4, LAG3, BCL-2, BAX, GLUT1, HK2, IL-6, IL-12, IL-10. TGF-beta, Rac1, and mTOR1). bEnrichment analysis using the Elsevier Pathway Collection, highlighting the involvement of target genes in cancer immune evasion mechanisms, including IDO1-mediated immune escape and effector T cell inactivation. c-d Comprehensive pathway and network analyses were performed to elucidate that the targeting genes are closely associated with immune response pathways and metabolic processes. Fig. S2.Aptamer-siRNA promotes apoptosis and inhibits proliferation in subcutaneous liver tumors.a Quantitative analysis of angiogenesis-related markers (VEGF, MMP-3, and ANG-1) by flow cytometry in tumours treated or not treated with aptamer-siRNA. b Ki67 staining to assess cell proliferation in tumour sections. Scale bar, 100µm. Data are presented as mean ± s.d. (n = 3 per group). c Representative TUNEL staining to analyses the proportion of apoptotic cells. Scale bar, 100µm. Statistical analysis was performed using one-way ANOVA with Tukey’s post hoc test.*P< 0.05, **P< 0.01, ***P < 0.001.Fig. S3.Aptamer-siRNA chimera treatment alters the number of Treg cells in tumours without affecting macrophage polarization. a Absolute numbers of total CD4⁺ T cells, Treg cells (FoxP3⁺CD4⁺), Th1 cells (IFN-γ⁺CD4⁺), and Th17 cells (IL-17A⁺CD4⁺) were determined by flow cytometry in healthy spleen, PBS-treated tumors, and chimera-treated tumors. b Flow cytometric analysis of tumour‑associated macrophage polarization showing no significant changes following aptamer-siRNA treatment. c-d Flow cytometry analysis of intratumoral CD3⁺CD8⁺ T cells in healthy, PBS-treated, and chimera-treated tumors, presenting both the proportion of CD8⁺ T cells among CD45⁺ cells and the absolute number per milligram of tumor tissue. e-g Quanti [file 43556_2025_392_MOESM1_ESM.docx]

**Targeting Tumor-Infiltrating Regulatory T Cells:
Combining CD47 and PD-L1 Inhibition via a novel aptamer-siRNA chimera**

Yu Zeng^1^*, Xiaoli Chen^1^*, Wenqiong Huang^1^*, Chi Ho Chan^2,3^*, Ziqi Chen^2,3^*, Minchuan Lyu^2,3^, Yumeng Liu^2,3^, Meijun Liu^1^, Aiping Lyu^1#^, Claudio Mauro^4#^, Yuanyuan Yu^2,3#^, Kenneth CP Cheung^*1#$^

**Author information**

^1^ Phenome Research Center, Hong Kong Baptist University, Hong Kong, China

^2^ Institute of Integrated Bioinformedicine and Translational Science (IBTS), School of Chinese Medicine, Hong Kong Baptist University, Hong Kong, China

^3^ Guangdong-Hong Kong-Macao Greater Bay Area International Research Platform for Aptamer-based Translational Medicine and Drug Discovery, Hong Kong, China

^4^ College of Medicine and Dental Sciences, University of Birmingham, Birmingham, UK

^*^Equal contributions

^#^ Corresponding authors: A Lyu: aipinglu@hkbu.edu.hk, C Mauro: c.mauro@bham.ac.uk, Yuanyuan Yu: yuyuanyuan@hkbu.edu.hk, Kenneth CP Cheung: kcpcheung@hkbu.edu.hk

^$^ Lead contact: kcpcheung@hkbu.edu.hk


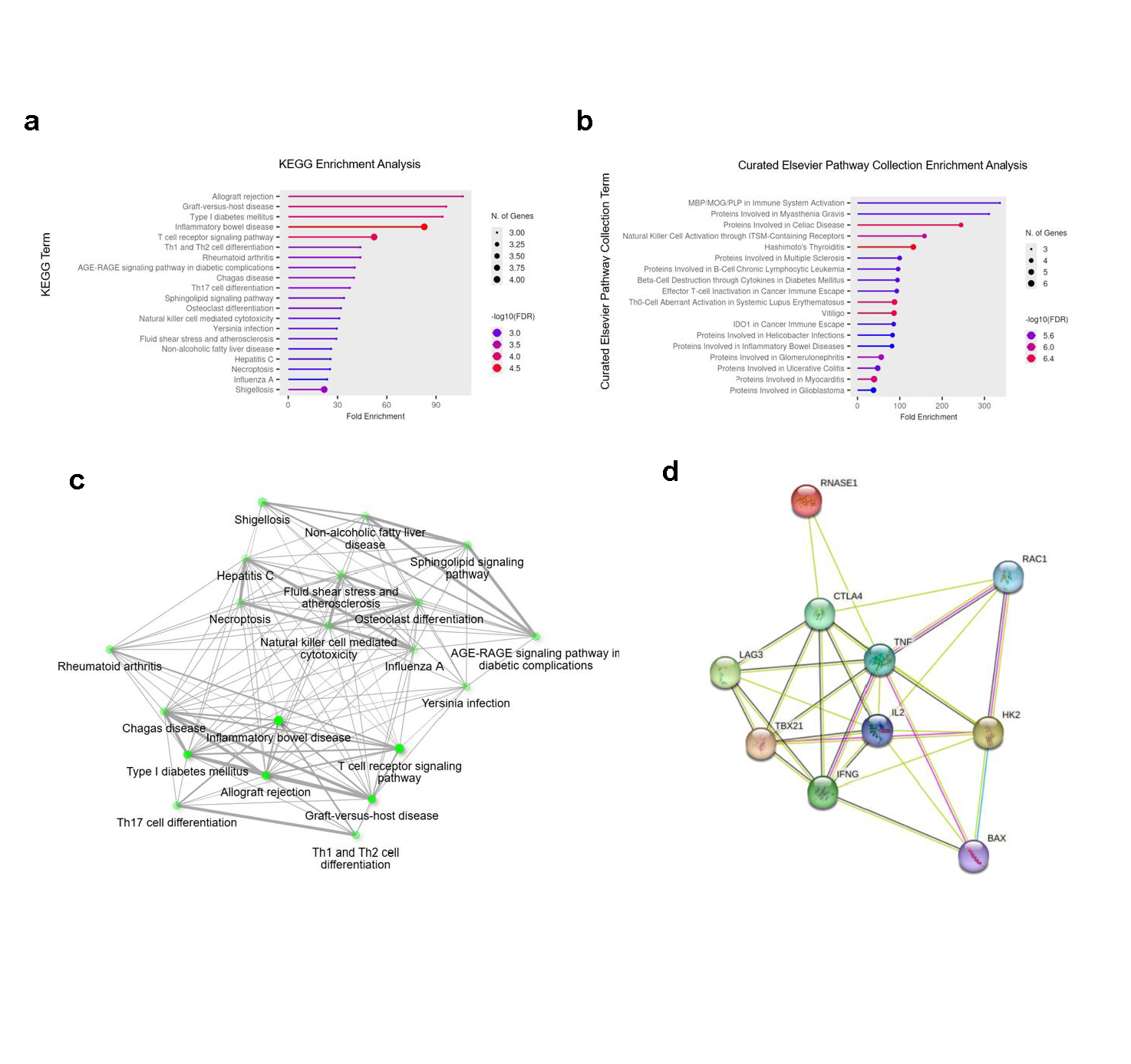


**Fig. S1 Pathway enrichment analysis of PD-L1- and CD47-associated genes. a** KEGG pathway enrichment of genes associated with PD-L1 and CD47 (IL2, IFNG, TNF, TBX21, EOMES, CTLA4, LAG3, BCL-2, BAX, GLUT1, HK2, IL-6, IL-12, IL-10. TGF-beta, Rac1, and mTOR1). **b** Enrichment analysis using the Elsevier Pathway Collection, highlighting the involvement of target genes in cancer immune evasion mechanisms, including IDO1-mediated immune escape and effector T cell inactivation. **c-d** Comprehensive pathway and network analyses were performed to elucidate that the targeting genes are closely associated with immune response pathways and metabolic processes.


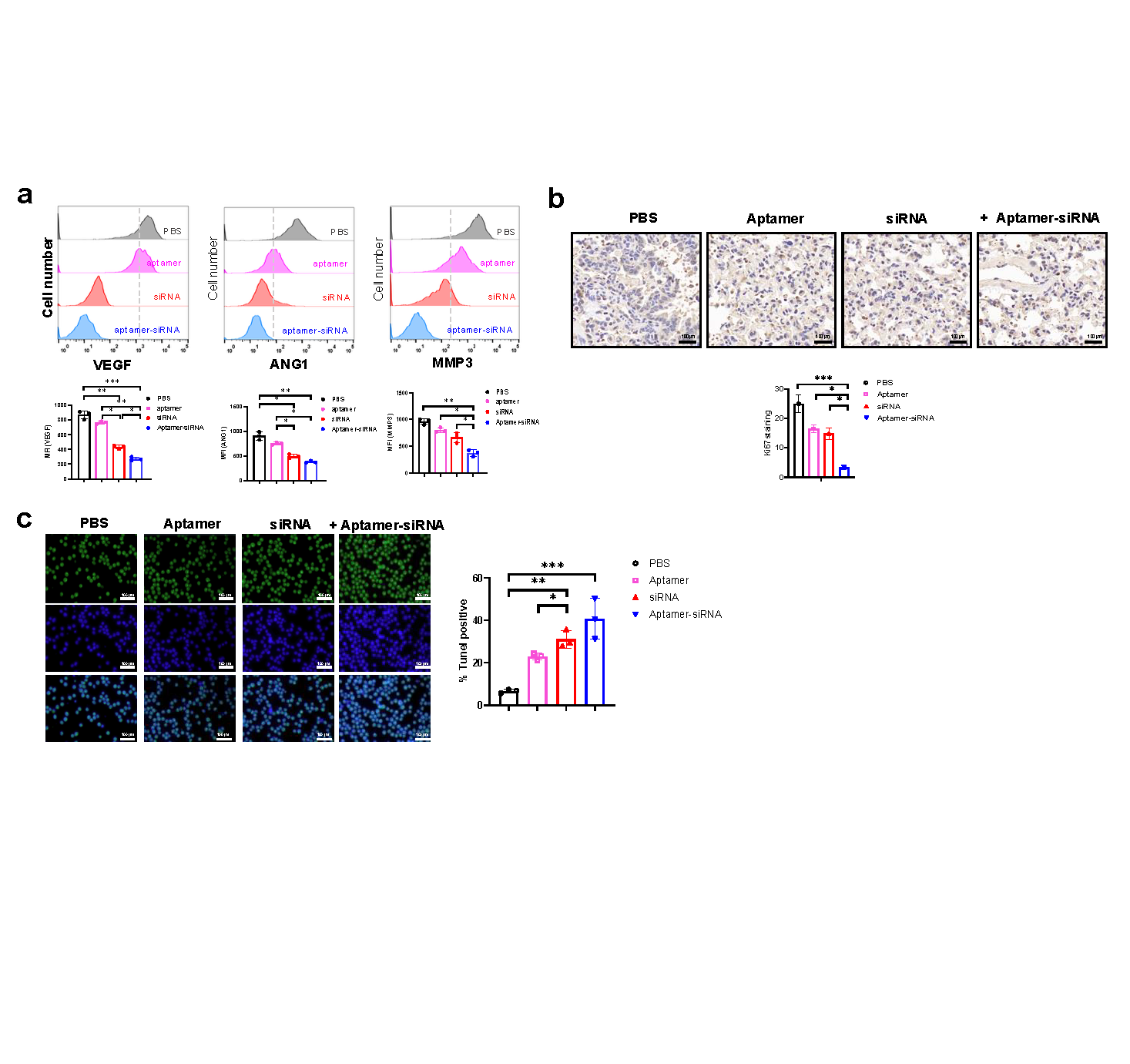


**Fig. S2 Aptamer-siRNA promotes apoptosis and inhibits proliferation in subcutaneous liver tumors. a** Quantitative analysis of angiogenesis-related markers (VEGF, MMP-3, and ANG-1) by flow cytometry in tumours treated or not treated with aptamer-siRNA. **b** Ki67 staining to assess cell proliferation in tumour sections. Scale bar, 100µm. Data are presented as mean ± s.d. (n = 3 per group). **c** Representative TUNEL staining to analyses the proportion of apoptotic cells. Scale bar, 100µm. Statistical analysis was performed using one-way ANOVA with Tukey’s post hoc test. **P <* 0.05*, **P <* 0.01*, ***P <* 0.001*.*


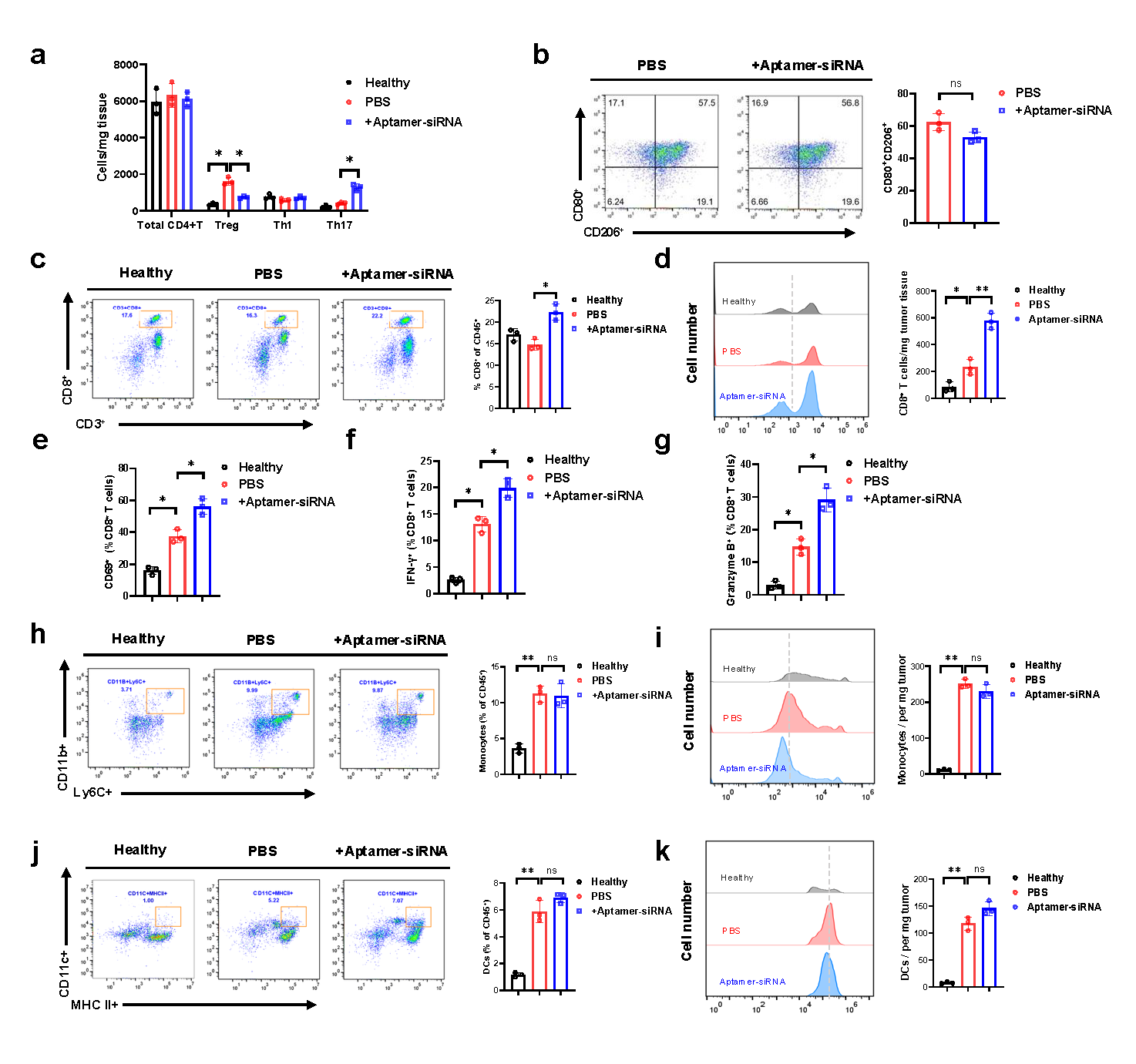


**Fig. S3 Aptamer-siRNA chimera treatment alters the number of Treg cells in tumours without affecting macrophage polarization. a** Absolute numbers of total CD4⁺ T cells, Treg cells (FoxP3⁺CD4⁺), Th1 cells (IFN-γ⁺CD4⁺), and Th17 cells (IL-17A⁺CD4⁺) were determined by flow cytometry in healthy spleen, PBS-treated tumors, and chimera-treated tumors. **b** Flow cytometric analysis of tumour‑associated macrophage polarization showing no significant changes following aptamer-siRNA treatment. **c-d** Flow cytometry analysis of intratumoral CD3⁺CD8⁺ T cells in healthy, PBS-treated, and chimera-treated tumors, presenting both the proportion of CD8⁺ T cells among CD45⁺ cells and the absolute number per milligram of tumor tissue. **e-g** Quantification of the proportion of (e) CD69⁺, (f) IFN-γ⁺, and (g) Granzyme B⁺ cells among total CD8⁺ T cells in tumor tissues.**h-i** Analysis of the frequency and absolute number of intratumoral monocytes following PD-L1×CD47 aptamer-siRNA chimera treatment.**j-k** Analysis of the frequency and absolute number of intratumoral dendritic cells (DCs) following PD-L1×CD47 aptamer-siRNA chimera treatment. Data are presented as mean ± s.d. (n=3 per group). Statistical significance was assessed using one-way ANOVA followed by Tukey’s multiple comparisons test or two-tailed Student’s t-test. ns, not significant; **P <* 0.05*，**P <* 0.01*.*


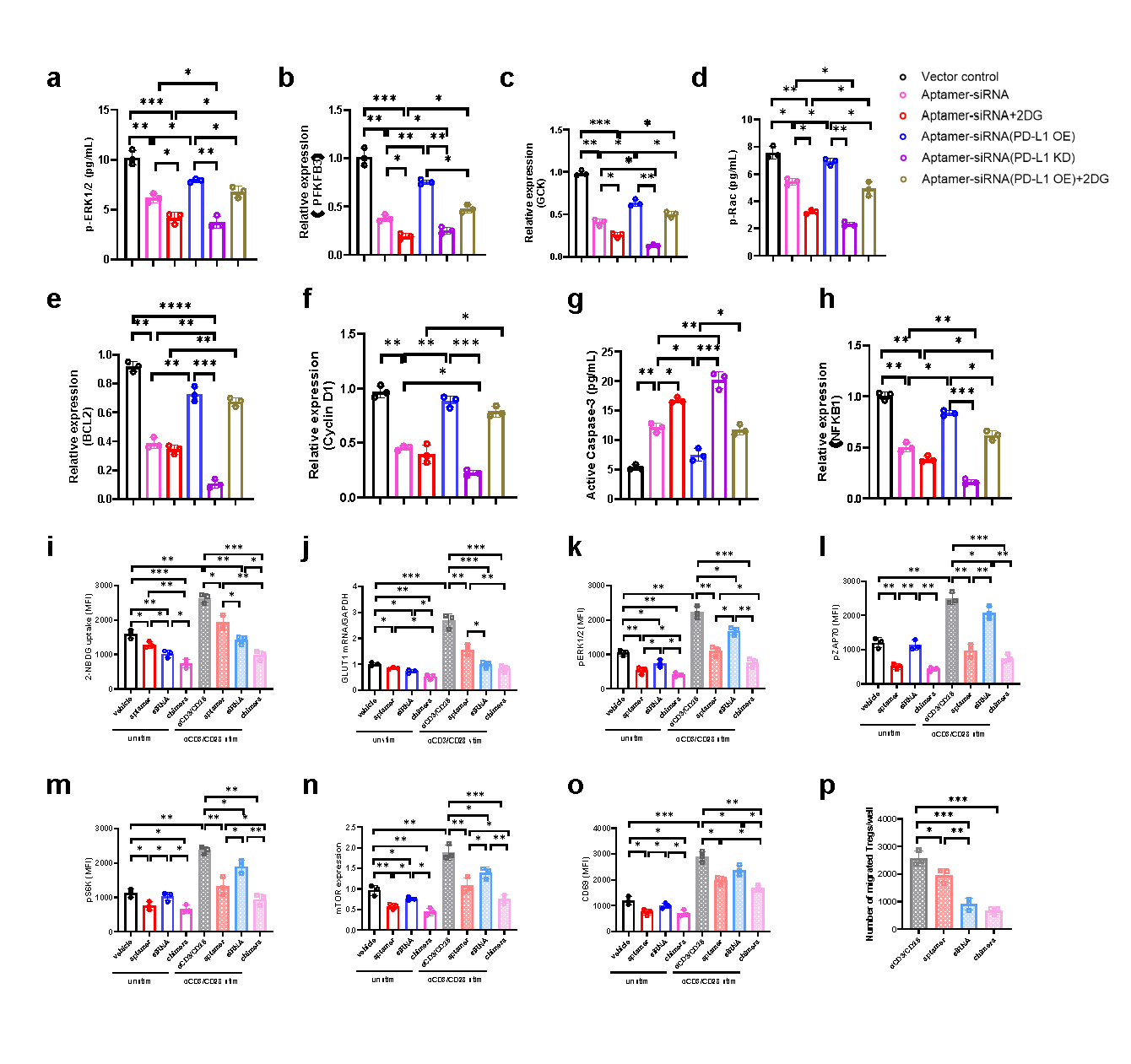


**Fig. S4 Effects of PD-L1 modulation and metabolic intervention on Treg cell function. a-h** Quantitative PCR analysis of PFKFB3, GCK, BCL2, CYCLIN D1 and NFKB1 mRNA levels, and ELISA measurement of p-ERK1/2, p-RAC and CASPASE3 protein expression in Treg cells across all groups. **i–j** Metabolic activity of tumor-infiltrating Tregs, assessed by (i) 2-NBDG uptake, and (j) GLUT1 expression, following aptamer-siCD47 treatment with or without TCR stimulation (αCD3/CD28). **k-o** Levels of (k) pERK1/2, (l) pZAP70, (m) pS6K, (n) mTOR, and (o) CD69 in tumor-infiltrating Tregs were quantified by flow cytometry or qPCR following aptamer-siCD47 treatment with or without TCR stimulation (αCD3/CD28). **p** Number of migrated Treg cells upon TCR stimulation with αCD3/CD28.Data are mean ± s.d. (n = 3 per group). Statistical analysis was performed using one-way ANOVA with Tukey’s post hoc test. **P <* 0.05*, **P <* 0.01*, ***P <*0.001*, ****P <* 0.0001.


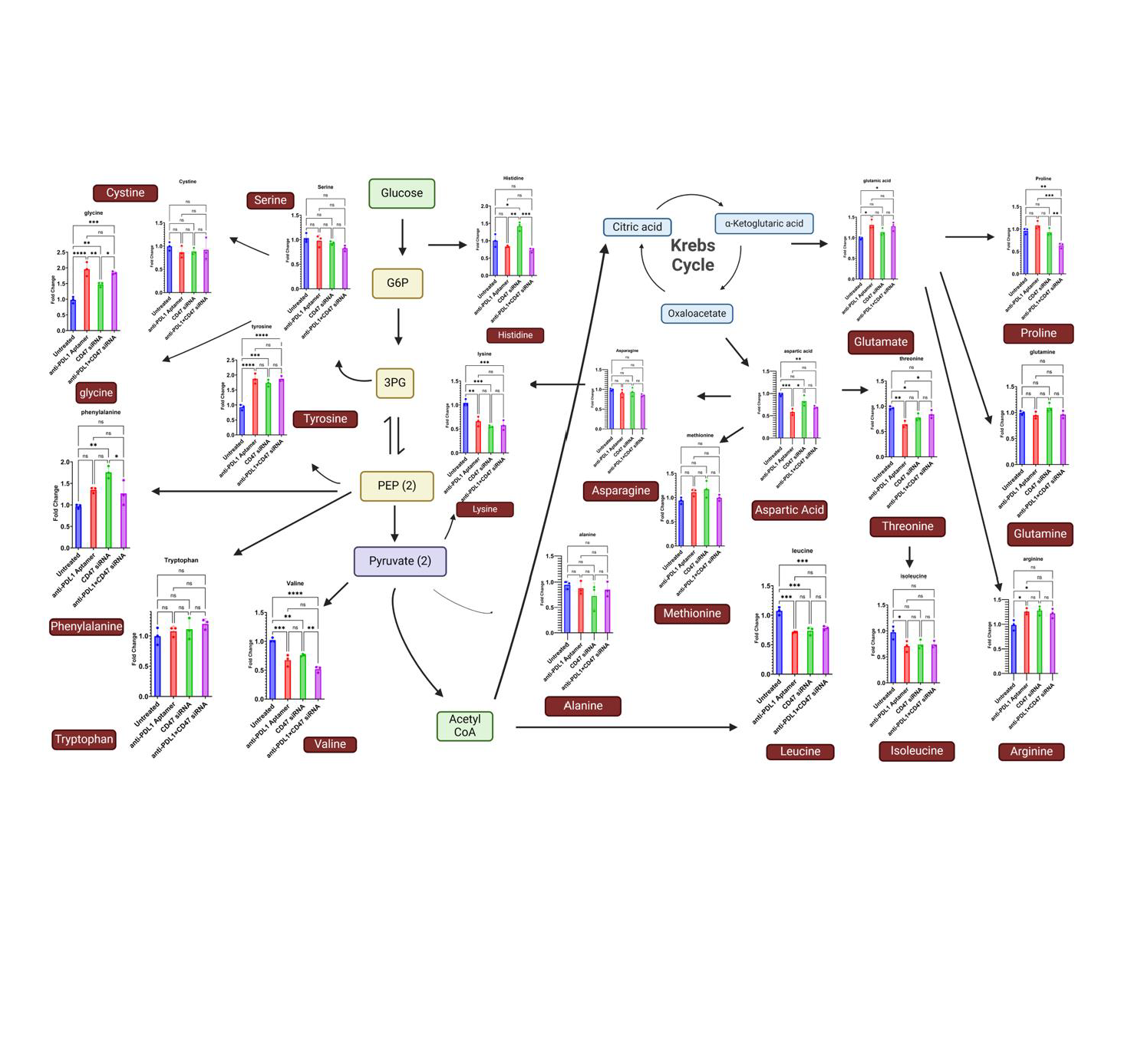


**Fig. S5 Amino acid metabolic enrichment analysis.** Amino‑acid levels in tumour‑infiltrating regulatory T cells isolated from liver tumours were measured following treatment with saline, anti‑PD‑L1 aptamer, CD47 siRNA or the aptamer-siRNA chimera. Data are presented as mean ± s.d. (n = 3 per group). Statistical significance was determined by Student’s t-test: **P<* 0.05*, **P <* 0.01*, ***P <*0.001*, ****P <* 0.0001.
